# An ELISA-based platform for rapid identification of structure-dependent nucleic acid–protein interactions detects novel DNA triplex interactors

**DOI:** 10.1016/j.jbc.2022.102398

**Published:** 2022-08-18

**Authors:** Nicholas G. Economos, Upasna Thapar, Nanda Balasubramanian, Georgios I. Karras, Peter M. Glazer

**Affiliations:** 1 Department of Therapeutic Radiology, Yale University School of Medicine, New Haven, Connecticut, USA; 2 Department of Genetics, Yale University School of Medicine, New Haven, Connecticut, USA; 3 Department of Genetics, The University of Texas MD Anderson Cancer Center, Houston, Texas, USA; 4 Genetics and Epigenetics Graduate Program, The University of Texas MD Anderson Cancer Center UTHealth Houston Graduate School of Biomedical Sciences, Houston, Texas, USA

**Keywords:** high-throughput screening, DNA structure, DNA repair, DNA binding protein, protein–DNA interaction, gene therapy, genome editing, ChIP, chromatin immunoprecipitation, dsDNA, double-stranded DNA, MS, mass spectrometry, PNA, peptide nucleic acid, SSA, single-strand annealing, ssDNA, single-stranded DNA, tcPNA, tail-clamp PNA, TFO, triplex-forming oligonucleotide

## Abstract

Unusual nucleic acid structures play vital roles as intermediates in many cellular processes and, in the case of peptide nucleic acid (PNA)–mediated triplexes, are leveraged as tools for therapeutic gene editing. However, due to their transient nature, an understanding of the factors that interact with and process dynamic nucleic acid structures remains limited. Here, we developed snapELISA (structure-specific nucleic acid-binding protein ELISA), a rapid high-throughput platform to interrogate and compare up to 2688 parallel nucleic acid structure–protein interactions *in vitro*. We applied this system to both triplex-forming oligonucleotide–induced DNA triplexes and DNA-bound PNA heterotriplexes to describe the identification of previously known and novel interactors for both structures. For PNA heterotriplex recognition analyses, snapELISA identified factors implicated in nucleotide excision repair (XPA, XPC), single-strand annealing repair (RAD52), and recombination intermediate structure binding (TOP3A, BLM, MUS81). We went on to validate selected factor localization to genome-targeted PNA structures within clinically relevant loci in human cells. Surprisingly, these results demonstrated XRCC5 localization to PNA triplex-forming sites in the genome, suggesting the presence of a double-strand break intermediate. These results describe a powerful comparative approach for identifying structure-specific nucleic acid interactions and expand our understanding of the mechanisms of triplex structure recognition and repair.

Nucleic acids are structurally polymorphic. Beyond the canonical B-DNA double helix model, nucleic acids adopt, often transiently, unusual structures that dynamically dictate critical life processes including DNA repair, replication, recombination, gene regulation, telomere protection, and nucleoprotein formation, among others (1, 2, 3, 4, 5). Unusual nucleic acid structures also play pivotal roles in the pathogenesis of diverse diseases, ranging from cancers to neurodegenerative disorders (6). Engineered synthetic nucleic acid analogues further expand the biochemical capabilities of nucleic acid structures by harnessing the powerful features of synthetic nucleic acid mimics with new attributes (7). Peptide nucleic acids (PNAs), for example, are chimeric oligonucleotides with a neutrally charged polypeptide backbone (8, 9). As a result of reduced repulsive forces between backbones, PNAs bind DNA and RNA with remarkably high affinity and specificity, allowing them to create novel artificial structures that remain stable within living cells. Notably, chemically modified PNAs designed to bind a target genomic DNA strand via Watson–Crick and Hoogsteen base-pairing form recombinogenic heterotriplex structures capable of catalyzing site-specific gene editing (10).

Despite their important contributions to fundamental processes and powerful applications as biotechnologies, methods to investigate factors that detect and process nucleic acid structures remain limited. The short-lived nature of structural intermediates often complicates their identification and investigation. To date, various approaches have been employed to study structure-dependent nucleic acid–protein interactions (11, 12). Current methods include *in vitro* techniques such as electrophoretic mobility shift assays and footprinting assays and biophysical techniques such as surface plasmon resonance. However, although informative and structure specific, these approaches lack throughput capacity and are best applied to describe specific interactions of interest. *In vivo*, chromatin immunoprecipitation (ChIP) and CRISPR-Cas9-based techniques offer useful insights into DNA–protein interactions and can be adapted for powerful high-throughput applications or coupled to mass spectrometry (MS) methods for protein identification (13). These techniques, however, measure interactions with DNA sequences within an entire population of cells and are agnostic to the three-dimensional nucleic acid structure. As a result, these indirect approaches may fail to distinguish transient structure-dependent interaction events or may report additional sequence interacting factors irrelevant to the structure of interest. Lastly, the aforementioned approaches tend to rely on large volumes of starting cellular material. *In vitro* methods overcome these limitations, although current available assays rely on sophisticated microfluidic setups and specialized bioinformatic pipelines (14). More readily accessible quantitative methodologies are needed for the *de novo* identification of structure-dependent interactions involving noncanonical or unstable nucleic acid structures in high throughput.

Given the important biological contributions of rare nucleic acid structures and a scarcity of tools to study their biology, strategies to investigate structure-specific protein interactions could provide valuable insights. Here, we describe snapELISA (structure-specific nucleic acid-binding protein ELISA), a high-throughput ELISA-based platform for rapid *in vitro* identification of structure-specific binders of nucleic acid assemblies. To circumvent the difficulties of observing transient structural interactions, we determined conditions to form and immobilize massively enriched stable nucleic acid structures and directly interrogate their recognition by a library of nucleic acid–binding factors. Applying this technique to triplex-forming oligonucleotide (TFO)–induced DNA triplex and artificial PNA:DNA:PNA heterotriplex structures measuring up to 2688 candidate interactions in parallel identified known and novel recognition factors for each structure. For PNA heterotriplexes, we go on to validate the identified interactions at therapeutically relevant loci in human cells. We thus present a flexible and convenient high-throughput method to resolve the interaction proteomes of noncanonical nucleic acid structures.

## Results

### snapELISA approach to identify nucleic acid structure–protein interactions

**Figure 1:**
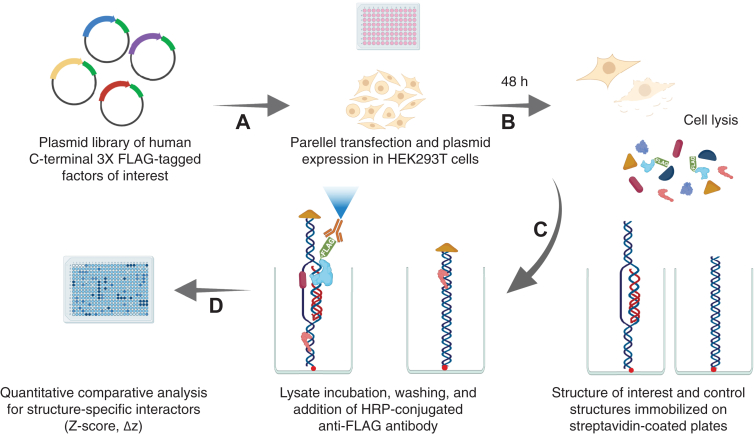
**Schematic of snapELISA approach**. *A*, HEK293T cells are transfected with a plasmid library of 3XFLAG-tagged factors of interest in 96-well format such that each well expresses a single factor. *B*, forty-eight hours later, transfected wells are washed and lysed to generate lysates containing expressed factors. Streptavidin-coated 384-well plates are separately coated with a biotin-conjugated structure of interest and a control structure. *C*, structure-bound plates are incubated in HEK293T cell lysates in parallel and washed to remove nonspecific interactors, and an HRP-conjugated anti-FLAG antibody and chemiluminescent substrate are added. *D*, structure–factor interaction signals are quantified by chemiluminescence detection and compared for significant differences between structures (Δz). HRP, horseradish peroxidase.

To systematically identify nucleic acid structure–protein interactions, we developed snapELISA as an unbiased method to screen for *in vitro* interactions between surface-immobilized nucleic acid structures and a tagged library of human proteins of interest. First, tagged libraries of C-terminal 3XFLAG-tagged plasmid-based constructs were expressed in HEK293T cells (Fig. 1*A*). To preserve any required binding partners and to reflect an intracellular-like context, tagged factors were introduced onto structure-bound platforms as whole-cell lysates and stringently washed after a 3-h incubation step (Fig. 1, *B* and *C*). Quantifiable detection of factor–structure interactions was then achieved by measuring chemiluminescent ELISA output after treating with horseradish peroxidase–conjugated anti-FLAG antibody (Fig. 1*D*). Parallel screening of libraries for structures of interest as compared to a relevant control structure (e.g., double-stranded DNA [dsDNA] of the same sequence) allows identification of meaningful interactors with specific affinity for the structure under study (Δz). The snapELISA approach is flexible and scalable, allowing for customizable protein library interrogation using any *in vitro* generated nucleic acid structure in high or low throughput. Here, we employ snapELISA to screen a small 90-factor library for TFO-induced DNA triplex interactors. We then expand this library to 524 human proteins, including 340 DNA repair factors, to find interactors of miniPEG γ-modified peptide nucleic acid–induced heterotriplexes (PNA:DNA:PNA) opposite a displaced single-stranded DNA (ssDNA) strand.

### snapELISA reliably detects structure-specific interactions for DNA triplexes

Active nucleic acid structures and intermediates forming within cells are particularly elusive to investigation by conventional means due to their highly dynamic nature. We envisioned that *in vitro* generation and immobilization of structures would create a more efficient and enriched context for investigation. Further, *in vitro*, aqueous conditions can be manipulated and optimized to stabilize specific structures of interest for direct characterization.

**Figure 2:**
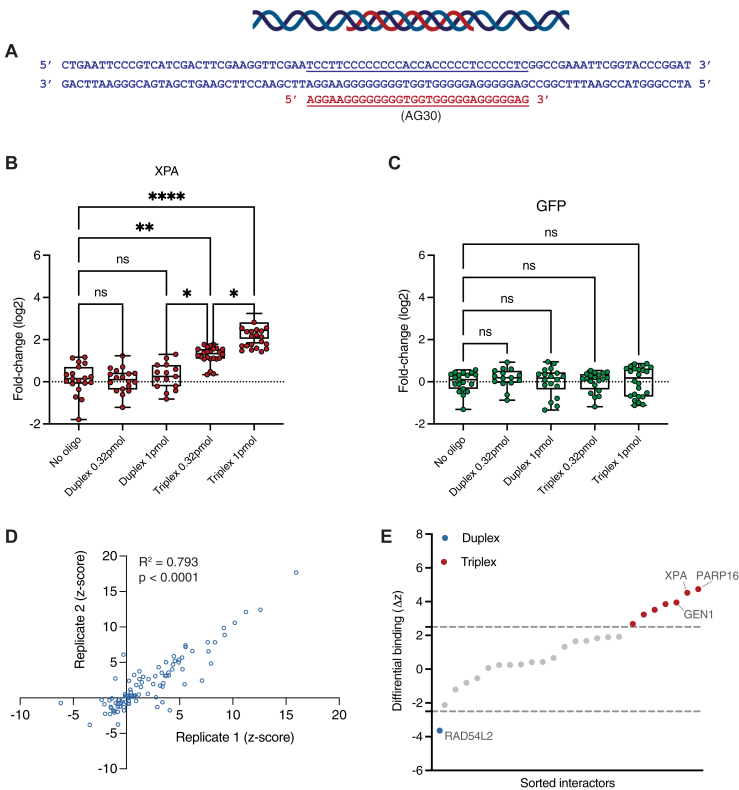
**snapELISA identifies TFO-induced DNA triplex interactors.***A*, schematic and sequences of oligonucleotides used to generate DNA triplex structures for snapELISA. Duplex DNA oligonucleotides (82 bp, in *blue*) coordinate with a triplex-forming DNA oligonucleotide (AG30, 30 nt, in *red*) to form a target triplex structure (underlined). *B*, fold-change (log2) in snapELISA signal for structures treated with XPA 3XFLAG-tagged whole-cell lysate by snapELISA methods. *C*, fold-change (log2) in snapELISA signal for structures treated with GFP 3XFLAG-tagged whole-cell lysate by snapELISA methods. *D*, replicate z-scores and correlation scoring for snapELISA screen of DNA triplexes. R^2^ and *p*-value are labeled on plot. *E*, waterfall plot representing differential binding (Δz) for snapELISA screen candidates. Hits with significant preference for duplex (*blue*) or TFO-induced triplex (*red*) structures are represented above and below Δz cutoff lines (Δz =2.5, Δz = -2.5). Select hits of interest are labeled. For *B* and *C*, box-and-whisker plots represent mean with 25th and 75th percentiles and maximum and minimum values from at least *n* = 15 independent experiments, *p*-values listed in Supplementary Table S3. For *D* and *E*, a screening library consisting of selected DNA repair-implicated factors from the Genome Maintenance Factor Library described by Karras *et al*. was used.

We first sought to construct DNA triplex structures featuring a TFO bound to a target double helix (Fig. 2*A*). Using published sequences and methods, we incubated a preformed biotin-conjugated 84-bp DNA duplex with a polypurine triplex–binding region (derived from the supFG1 gene) and a corresponding target 30-nt TFO (AG30) to form stable DNA triplexes (15). Previous studies have identified factors implicated in the recognition and resolution of TFO-induced structures and thus act as convenient validation candidates for snapELISA experiments. As a proof of principle for the snapELISA approach, we employed the known TFO-induced triplex recognition factor and nucleotide excision protein XPA (16, 17). In these experiments, two quantities (0.32 pmol and 1 pmol) of duplex and TFO triplex structures were immobilized to the surface of a streptavidin-coated plate and incubated with lysate of HEK293T cells overexpressing either 3XFLAG-tagged XPA protein or, as a control, 3XFLAG-tagged GFP (green fluorescent protein, no expected interaction). Specific recruitment of FLAG-tagged proteins to the immobilized nucleotide structures was determined by snapELISA methods and z-score calculation.

As expected, snapELISA detected a dose-dependent increase in signal above baseline when XPA lysates were incubated with TFO triplex structures (*p* = 0.0010, *p* < 0.0001 for 0.32 pmol and 1 pmol, respectively, Fig. 2*B*). In contrast, GFP-expressing lysates incubated with TFO structures produced no significant snapELISA signal, which was comparable to the GFP and XPA background “no oligo” control conditions (*p* > 0.99 across conditions, 2C). Importantly, neither GFP nor XPA was recruited to duplex structures (*p* > 0.99 across conditions, Fig. 2, *B* and *C*). These results demonstrate that snapELISA methods can reliably detect DNA triplex–specific interactions *in vitro* with low nonspecific background signal.

Finally, using optimized snapELISA protocols for TFO-induced triplex structures, we applied a small-scale screen to interrogate a 90-factor library for TFO triplex–specific interactors. Our expression library featured DNA repair–implicated factors selected from a library generated and previously described by Karras *et al*. (18). snapELISA screening was applied to both target TFO triplex structures and control duplex structures to determine binding signals and z-scores relative to background (empty) wells within each plate. Additionally, 3× FLAG-tagged GFP lysates were included in each plate as negative screen controls. Screens were conducted in biological duplicate for each structure, and correlation scores between replicates suggested strong reproducibility (R^2^ = 0.793, *p* < 0.0001, Fig. 2*D*). GFP controls demonstrated negligible ELISA signal with no detected binding differences between structure conditions (data not shown). Ultimately, using a z-score ≥ 5 and Δz ≥ 2.5 cutoff, snapELISA methods identified 7 of 90 total factors with specific binding to triplex but not duplex structures. Expected TFO triplex–specific interactor XPA was identified (Fig. 2*E*). Interestingly, we also observed novel hits including endonuclease GEN1 and PARP16 (Fig. 2*E*). However, TFO triplex–specific interactions were substantially weaker and less well correlated than duplex interactions, suggesting reduced homogeneity or thermodynamic stability of the triplex structures (Supplementary Fig. S1). Indeed, studies have shown the predicted melting temperature (T_m_) of a bound TFO is often substantially lower than that of its target DNA duplex (19). This observation prompted us to examine more stable structures using high-affinity triplex-forming PNAs.

### snapELISA scaling and application to synthetic PNA heterotriplex structures

**Figure 3:**
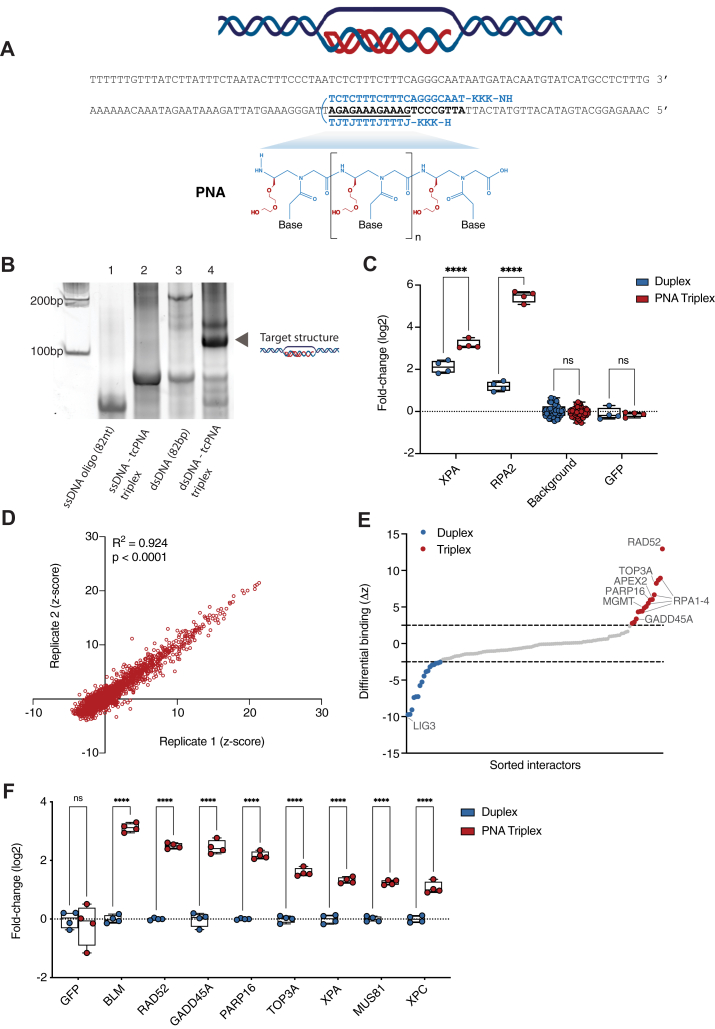
**snapELISA identifies PNA heterotriplex interactors.***A*, schematic and sequences of oligonucleotides used to generate PNA heterotriplex structures for snapELISA. Duplex DNA oligonucleotides in *black* (82 bp). A tail-clamp γMP-modified tcPNA molecule (in *blue*) coordinates with a target sequence (*bold*) to form a PNA:DNA:PNA heterotriplex opposite a displaced ssDNA strand. γMP PNA structure is highlighted below, with γ-polyethylene glycol sidechains highlighted in *red* (J = β-d-glucopyranosyloxymethyluracil, K = L-lysine residue). *B*, triplex study structures on polyacrylamide gel. Each structure was assembled in streptavidin-coated wells, released by UV light exposure, and visualized using native-PAGE techniques. Target PNA heterotriplex band is labeled. *C*, fold-change (log2) in snapELISA signal for duplex (*blue*) and triplex (*red*) structures treated with XPA, RPA2, background (no FLAG), and GFP 3× FLAG-tagged whole-cell lysate by snapELISA methods. *D*, replicate z-scores and correlation scoring for all replicates in snapELISA screen of PNA heterotriplexes. R^2^ and *p*-value are labeled on plot. *E*, waterfall plot representing differential binding (Δz) for snapELISA screen candidates. Hits with significant preference for duplex (*blue*) or PNA heterotriplex (*red*) structures are represented above and below Δz cutoff lines (Δz =2.5, Δz = -2.5). Hits of interest are labeled on plot. *F*, snapELISA validation experiments using selected N-terminal 3× FLAG-tagged lysates. Each point is graphed as fold-change (log2) in snapELISA signal relative to averaged duplex condition value for each factor. For *C* and F, box-and-whisker plots represent mean with 25th and 75th percentiles and maximum and minimum values from at least *n* = 4 independent experiments, *p*-values listed in Supplementary Table S3. For D and E, a custom screening library generated for this study, as described in Experimental procedures and summarized in Supplementary Table S2, was used.

Next, we sought to apply snapELISA methods to identify interactors for PNA:DNA:PNA heterotriplex structures formed by a tail-clamp PNA (tcPNA) that binds to one strand of a target DNA duplex via both a Watson–Crick binding domain and a Hoogsteen binding domain (Fig. 3*A*) (20, 21). Such “clamped” PNA heterotriplexes opposite a displaced DNA strand are dramatically more stable than TFO-induced triplex structures, which greatly facilitates site-specific gene editing when targeted to genomic loci (22, 23). While these synthetic nucleic acid structures have been demonstrated as biotechnologies capable of ameliorating disease phenotypes in mouse disease models via *ex vivo*, *in vivo*, and *in utero* gene editing, the mechanisms by which endogenous proteins recognize PNA heterotriplexes remain to be fully elucidated (24, 25, 26, 27). Thus, PNA heterotriplexes present an attractive candidate for interrogation by snapELISA methods to further understand and potentially improve their rational design and application.

As we did for the TFO-based snapELISA screen, we generated PNA heterotriplex structures *in vitro* and subsequently immobilized them to a streptavidin-coated plate. For this screen, we chose to investigate PNA heterotriplexes in a duplex DNA context featuring an extruded single DNA strand to emulate recombinogenic structures that would be encountered in chromatin within living cells (Fig. 3*A*). For this study, we used commercially coated plates and a miniPEG γ-modified tail-clamp peptide nucleic acid (γMP-tcPNA) with a 20-nt Watson–Crick binding portion and an antiparallel 12-nt triplex-forming Hoogsteen binding portion bound to an 82-bp DNA target sequence (Fig. 3*A*, Supplemental Figs. S2 and S3) (26). The sequences of these molecules reflect clinically relevant targets previously used to correct a pathologic beta-thalassemia-associated splicing mutation in the human *HBB* (hemoglobin subunit beta) gene in transgenic mice (26). To generate structures *in vitro* and maximize target structure enrichment, we introduced each nucleic acid moiety to streptavidin-coated plates in a stepwise approach. Initial experiments were conducted using a photocleavable biotin conjugate to allow structure release from plates after UVA wavelength exposure. Native-PAGE analysis was used to confirm optimized annealing conditions after each step (Fig. 3*B*). We first introduced a biotinylated ssDNA oligo (82 nt, Fig. 3*B* – lane 1) and a triplex-forming tcPNA oligomer to form a stable and immobilized heterotriplex structure (Fig. 3*B* – lane 2). Next, a complementary ssDNA oligo (length: 82 nt) was added to bind the remaining exposed DNA bases and generate a duplex context for the formed triplex structure to approximate genomic DNA invasion events (target structure, Fig. 3*B* – lane 4). Native-PAGE analysis revealed shifted bands for each expected structure, suggesting a strong enrichment for target structures generated using this stepwise approach (Fig. 3*B*). In the case of the heterotriplex target structure (Fig. 3*B* – Lane 4), a second band is observed and represents an expected additional minor triplex structural variant. These observations are consistent with prior studies investigating diversity of *in vitro* generated PNA triplex invasion structures (28, 29).

We confirmed PNA heterotriplex structure-specific binding by snapELISA using two known heterotriplex interacting factors, XPA and RPA2 (30). As expected, snapELISA signals for XPA- and RPA2-treated PNA heterotriplexes were significantly greater than for XPA- and RPA2-treated duplexes (Fig. 3*C*, *p* < 0.0001, across conditions). Specifically, XPA and RPA showed 2- and 20-fold preference in binding to PNA heterotriplexes as compared to DNA duplex structures, respectively. In contrast, signals for background (no FLAG-protein) and GFP-treated structures were low and nonsignificantly differing (Fig. 3*C*, *p* > 0.4 across conditions). Further, to explore any potential impact of the number of cells transfected on snapELISA performance, we conducted additional experiments comparing PNA heterotriplex binding of GFP, XPA, and RPA2 across different seeded cell concentrations. We noted similar trends across conditions, suggesting increased protein abundance in lysate inputs did not affect the observed results (Supplemental Fig. S4). These results demonstrate the ability of snapELISA approaches to detect structure-specific protein interactions with low background using a second structurally distinct platform.

We next applied high-throughput snapELISA methods to PNA heterotriplexes using a custom designed library of 520 human proteins (Supplementary Table S2). This library was designed to include 336 unique DNA repair proteins and splicing variants (289 DNA repair genes). As controls, we utilized 184 human proteins with no known functions in DNA repair, 64 of which are chromatin associated (Supplementary Table S2). Screening was performed in biological duplicates for PNA heterotriplexes and control duplex structures in 384-well plates. Correlation scoring between replicates demonstrated excellent reproducibility across the screen (R^2^ = 0.924, *p* < 0.0001, Fig. 3*D*). Correlation scoring between replicates was especially improved for the PNA heterotriplex–specific interactors (R^2^ = 0.930, *p* < 0.0001, Supplemental Fig. S5). Next, preferential binding to PNA triplex structures was determined by calculating the difference in snapELISA signal between triplex and duplex controls using z-score ≥ 5 and Δz ≥ 2.5 cutoffs. Of 520 factors screened, we found that 126 were recruited to the triplex and/or duplex structures (24.2%). As expected, the frequency of interactors was significantly reduced across control proteins with no known chromatin association (12.5%, *p* = 0.00018, hypergeometric distribution test), as compared to chromatin-associated proteins with no known function in DNA repair or DNA repair factors (triplex *p =* 0.0051, duplex *p* = 0.0006, Supplementary Fig. S6). Ultimately, 16 proteins demonstrated a significant preference for PNA heterotriplex structures, while 17 demonstrated duplex preference (Fig. 3*E*, detailed results in Supplemental Table S2, Supplemental Fig. S7). The remaining 93 hits did not demonstrate a significant preference for either structure. As expected, ssDNA binding factor RPA2 was identified among the PNA heterotriplex-selective hits. In addition, the remaining 3 subunits of RPA, RPA1, RPA3, and RPA4 were also identified as strong triplex binders, which suggests RPA recruitment to the expected displaced ssDNA moiety within the study structure (Fig. 3*E*). However, XPA was a weak hit in the screen because the construct yielded poor expression results, as compared to the original plasmid we used for the aforementioned control experiments (Supplemental Fig. S8). Interestingly, snapELISA identified additional related and novel selective PNA heterotriplex–binding factors. Among these proteins were DNA excision factors APEX2, and GADD45A (Fig. 3*E*). Notable novel factors include strong hits for topoisomerase TOP3A, PARP16 mono-ADP-ribosyltransferase, and MGMT methyltransferase, implicating pathways related to structure-specific regulation of recombination and replication, and RAD52, implicating the single-strand annealing subpathway of homology-directed repair (HDR) (31).

Finally, interesting hits including RAD52, PARP16, GADD45A, and TOP3A were subjected to repeat follow-up validation experiments for reproducible confirmation (Fig. 3*F*). Indeed, all hits were reproducibly validated by snapELISA methods which were performed using clones harboring the 3XFLAG-tag at the opposite end of the protein as compared to the initial screen (N-terminus). Next, we interrogated additional hit-related DNA repair factors that were not identified by our original screen including BLM (bloom nuclease, TOP3A co-factor) and MUS81 and XPC (nucleotide excision repair factors). XPA was also included. All factors interrogated reproducibly demonstrated strong preferences for binding PNA heterotriplex structures (*p* < 0.001 across conditions, Fig. 3*F*). As a control, GFP produced no significant signal difference between structures by snapELISA (*p* > 0.8, Fig. 3*F*). Thus, low-throughput opposite-tagged validation experiments orthogonally confirmed significant binding preferences for factors identified by snapELISA screening. We further present evidence of strong triplex binding preference for related cofactors that were newly identified here using snapELISA.

### snapELISA hits localize to structure target sequences in human cells

**Figure 4:**
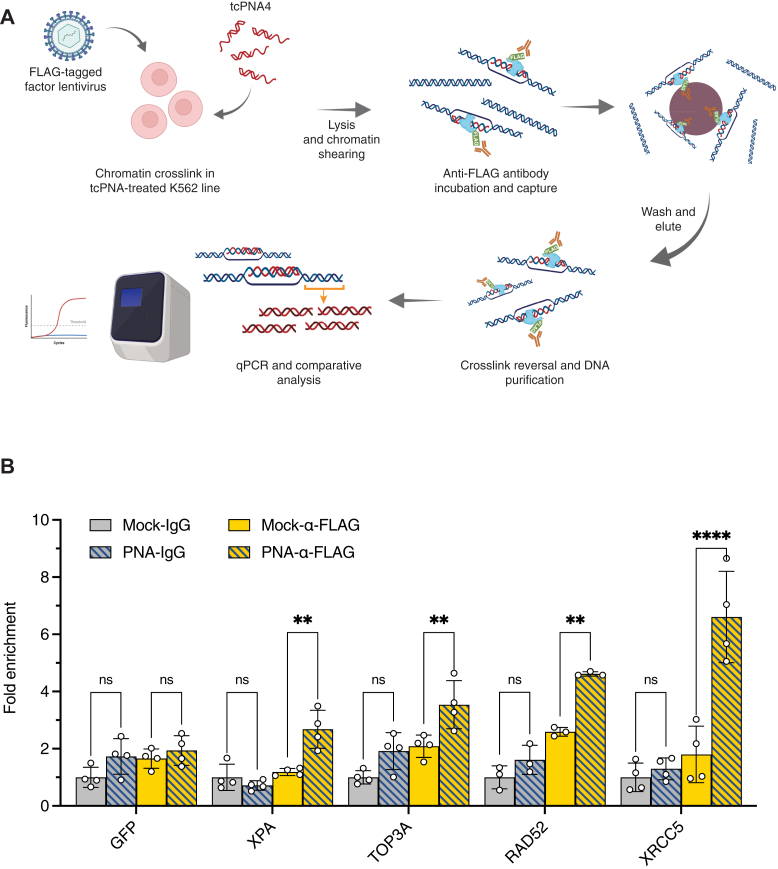
**snapELISA identified interactors localize to PNA-binding sites in human cells.***A,* schematic of chromatin immunoprecipitation-qPCR approach to identifying factor localization to PNA-formed structures in K562 cell lines. K562 cell lines were stably transduced with lentivirus expressing a FLAG-tagged factor and nucleofected with tcPNA4. 12 h later cells were cross-linked and lysed, and chromatin was sheared. Anti-FLAG antibody capture and bead purification provided final substrates for ChIP-qPCR analyses. *B,* ChIP localization of FLAG-tagged factors to tcPNA genomic-binding site. Each point is graphed as fold enrichment in signal relative to averaged mock condition. Bars represent ChIP experiments using mock (solid) and PNA-nucleofected (hatched) K562 cells treated with control IgG (gray) or anti-FLAG (yellow) antibody. For B, bars represent average measurements for 3 technical replicates from at least *n =* 3 independent experiments, mean differences and *p*-values are listed in Supplementary Table S3.

While *in vitro* investigations offer high throughput and convenient insights into structure–protein interactions, we next wanted to corroborate validated snapELISA hits by observing their in-cell interactions at genomic target loci. To do this, we sought to investigate whether protein hits localized to the therapeutically relevant HBB-IVS2 tcPNA binding site in human cells. For these experiments, human myeloid lineage K562 cell lines were generated to stably express 3XFLAG-tagged factors of interest. Stable cell lines were generated for GFP, XPA, TOP3A, RAD52, and XRCC5. For each factor line, cells were nucleofected with tcPNA to allow PNA-induced triplex formation with the genomic target sequence at the endogenous *HBB* locus (Fig. 4*A*). Subsequently, we evaluated the recruitment of FLAG-tagged proteins to the structure-forming target site within the nucleofected cells by chromatin immunoprecipitation and qPCR (ChIP-qPCR, Fig. 4*A*). Experiments revealed specific recruitment of XPA, TOP3A, and RAD52 to the genomic target site only in tcPNA-treated cells, as compared to vehicle-treated cells (compare PNA- α-FLAG *versus* Mock-α-FLAG, Fig. 4*B*). In contrast, we observed no significant protein recruitment by ChIP-qPCR using IgG control in place of α-FLAG in PNA- or vehicle-treated cells (Mock-IgG *versus* PNA-IgG, Fig. 4*B*). In initial snapELISA assays, we detected strong binding signals for the nonhomologous end joining factor XRCC5 (Ku80) to both duplex control and PNA heterotriplex structures (Z-score > 15). This observation was presumed due to binding to dsDNA blunt ends in both artificial substrates since it is well established that XRCC5 readily binds at DNA double-strand breaks (DSBs). Based on this, we included ChIP for XRCC5 as a control, anticipating a lack of blunt-end structures in the genomic context. We saw no localization of XRCC5 to the target genomic site in non-PNA-treated cells, as expected (Fig. 4*B*). Surprisingly, however, we observed significant XRCC5 recruitment to the location of the PNA-binding site after treatment, suggesting the presence of a blunt-ended DSB intermediate (Fig. 4*B*). Importantly, protein recruitment to PNA-based structures was specific to the target locus as PNA treatment did not promote the recruitment of XPA, TOP3A, RAD52, or XRCC5 to an off-target genomic site (Supplementary Fig. S9). These experiments establish the ability of snapELISA to detect structure-specific interactors relevant to the intermediates forming on chromatin bound by triplex-forming PNAs within living cells.

## Discussion

Nucleic acid structural intermediates are transient and thus elusive to many conventional biochemical methods of investigation. Similarly, synthetic nucleic acids have eluded thorough investigation of their genome editing capabilities and further rational advancement of their biotechnological applications. In response to these challenges and to improve understanding of rare structure recognition and repair, we report snapELISA as a convenient and customizable approach for low- or high-throughput interrogation of *in vitro* protein–nucleic acid structure interactions. As applied to TFO-induced DNA triplex and PNA:DNA:PNA heterotriplex structures, we demonstrate the ability of snapELISA to detect known and previously unknown interacting factors.

Our approach circumvents two notable difficulties of nucleic acid structure investigation. Firstly, overcoming the transitory or unstable nature of some nucleic acid structures, our *in vitro* approach allows users to optimize buffer, salt, and assembly conditions for temporally stable structures for observation. For example, in the case of PNA heterotriplexes in the context of dsDNA, sequential introduction of each nucleic acid component in optimized buffer conditions accommodated minimized alternative binding events to generate maximally enriched study structures (Fig. 3*B*). Secondly, to amplify signal and improve detection sensitivity, snapELISA methods employ platform-immobilized structures at high concentrations and featured 3XFLAG-tagged factor libraries for high-affinity antibody-mediated detection. Together, these strategies allow the sensitive and unbiased identification of otherwise transient interaction events that would be difficult to efficiently observe in endogenous contexts.

snapELISA presents important advantages over alternative approaches such as low-throughput *in vitro* biochemical assays (electrophoretic mobility shift assay, footprinting, etc.) and ChIP methods that focus on sequences rather than structures. Instead, snapELISA combines advantageous features from both assay types by using enriched *in vitro* structure formation coupled with high-throughput screening methods and expressed factors within whole-cell lysates. Although snapELISA enables rapid, quantitative, and comparative analyses of nucleic acid structure interactions, it does not consider the timing or genomic contexts of these interactions. Further, by nature of its *in vitro* and systematic design, snapELISA methods primarily focus on structure recognition and likely represent the proximal factors and stages of processing. Thus, the timing for factor recruitment is difficult to resolve with this approach. However, some time-resolved information may be inferred by using synchronized cell populations, such as after HU or thymidine block release, or in combination with fluorescence-activated cell sorting using G2/M- or G1-expressed fluorescent proteins in future experiments. An additional limitation of snapELISA is the difficult-to-predict effect of end-tagging on protein binding, stability, and activity. In our analysis, we noted interactors (BLM, MUS81, XPA, and XPC) that were not revealed by our C-terminal FLAG-tagged screen, but later analyses using N-terminal tagged constructs demonstrated robust differences (Fig. 3*F*). Conversely, other orthogonally validated hits, such as RAD52, were not as robust in N-terminal analyses as in C-terminal (5.66-fold *versus* 13-fold preference for PNA triplex binding, respectively). In the case of XPA from our PNA heterotriplex screen, low expression likely contributed to a weaker-than-anticipated signal (Supplemental Fig. S8). Thus, the relative strength of hits may not necessarily reflect relative importance and negative findings may be difficult to interpret. Future comprehensive screens may consider screening libraries featuring both end tags or, as in this study, identifying cofactors related to hits for further follow-up investigation. Additional strategies could aid in understanding expression or stability effects in the form of low-throughout Western blotting or Renilla luciferase-tagged bait proteins to measure bait expression in cell lysates in high throughput. The latter, however, would require construction of a different bait expression library involving Renilla luciferase in place of a 3XFLAG tag. Despite limitations, snapELISA provides a versatile method to evaluate the recruitment of proteins to a diverse array of nucleic acid structures.

Importantly, orthogonal ChIP-qPCR validation of snapELISA hits strongly suggests that the repair intermediates we examined *in vitro* are highly representative of the structures that form within living cells. In this study we use snapELISA to interrogate interactions for PNA-mediated triplex structures known to induce site-specific gene editing. Unlike CRISPR-Cas systems and nuclease-based gene editing platforms, PNA-mediated editing relies on the formation of synthetic heterotriplex structures with genomic DNA to trigger endogenous repair and facilitate site-specific recombination. Notably, this process occurs with less off-target activity compared to nuclease/DSB-mediated approaches (25). Understanding the repair factors processing these structures may help reveal novel mechanisms of gene editing and provide rational strategies to increase the efficiency of HDR-mediated repair. In this study, snapELISA screening implicates specific and novel pathways of interest for gene editing including nucleotide excision repair (XPA, XPC), the single-strand annealing (SSA) subpathway of HDR (RAD52), and factors associated with the resolution of recombination structural intermediates (TOP3A, BLM, MUS81, GADD45A). Interestingly, we also present data suggesting the presence of a blunt-ended DSB intermediate at the PNA triplex-forming target site (XRCC5, Fig. 4*B*). In line, previous work has shown that the MUS81 nuclease cleaves a variety of looped structural intermediates and, in concert with BLM helicase, is required to induce DNA DSBs in response to replication stress (31, 32, 33). Supported by *in vitro* snapELISA, we postulate triplex structure formation may generate recombinogenic DSB intermediates by causing DNA replication stress. Future studies may further elucidate which moieties within the PNA heterotriplex and displaced DNA strand are recognized by factors and the contribution of these pathways. For example, comparative screens for a single DNA strand with a PNA triplex around a strand of DNA could delineate factors that recognize the core triplex as opposed to the extruded single DNA strand.

Further comparing our snapELISA results for PNA structures to other screens for repair outcomes from the literature reveals differences across gene editing systems. Previous studies using Cas9- and Cas12a-induced DSBs with sequencing allowed authors to systematically map mechanisms of DSB repair with and without ssDNA oligonucleotides for HDR (34). While the identified hits were comparable to our PNA triplex selective binders, certain pathways related to homologous recombination were discordant between screens. SSA repair (RAD52), BLM/TOP3A-mediated regulation, and nucleotide excision repair (XPA, XPC) factors were absent from the dependencies observed by Hussman *et al*. In addition, central hits from their repair analysis knockout screens were not observed in our snapELISA-based screens, such as DSB processing MRN complex hits (MRE11, NBN, RAD50) and Fanconi anemia pathway proteins (34). Differences between these two conceptually distinct types of screens likely reflect important differences in the repair of PNA triplexes, which are also able to induce site-specific recombination with ssDNA templates, as compared to nuclease-induced DSBs. Overlaying data from genetic-based knockdown screens in PNA-treated cells with our *in vitro* snapELISA results may offer additional insights into the mechanisms by which PNA triplexes are repaired and induce site-specific editing. Interestingly, recent work demonstrated that artificial recruitment of SSA-pathway HDR factor RAD52 to DSBs improves CRISPR-Cas9 mediated editing; this target was not detected by the knockdown approach of Huissman *et al*. Studies co-expressing RAD52 or using RAD52-Cas9 fusion proteins in human cells demonstrated enhanced template-mediated HDR and a reduction in mutagenic nonhomologous end joining–mediated repair (35, 36). Considering our study, RAD52 fusion to triplex forming PNAs may prove to be an effective rational approach to improve HDR-mediated genome editing. Ultimately, the exact contributions and possible crosstalk between pathways implicated in this study remain to be elucidated. Future studies may inform approaches to favor recombinogenic repair pathways and improve efficiency.

Moreover, snapELISA provides a highly versatile methodology for future studies to elucidate the mechanism of nucleic acid recognition and processing for diverse DNA and RNA-binding proteins, including DNA repair factors, as well as a high-throughput functional genomics platform to characterize variants of uncertain clinical significance in these proteins. Finally, snapELISA can be easily paired with fluorescence techniques and other methodologies, such as IncPRINT (37), to allow multiplexed reading of protein or nucleic acid recruitment to enable highly sophisticated applications in functional genomics and drug screening.

In summary, we describe and validate snapELISA as a method to rapidly investigate the protein interactomes of otherwise elusive nucleic acid structures. As a demonstration we screened and identified novel factors implicated in the recognition of PNA heterotriplex structures, suggesting potential new mechanisms of interest for applications to therapeutic gene editing.

## Experimental procedures

### Cell culture

K562 cells (CCL-243, ATCC) were maintained in RPMI-1640 medium supplemented

with 10% fetal bovine serum (FBS, Life Technologies). HEK293 T cells (CRL-3216, ATCC) were maintained in DMEM medium supplemented with 10% fetal bovine serum. All cell lines were tested and confirmed to be free of *Mycoplasma* infection (Lonza MycoAlert) and were authenticated by STR profiling.

### Cell lines

3XFLAG-tagged stable K562 lines were established by lentiviral transduction using viral particles generated from C-terminal 3XFLAG-tagged cDNA cloned into a pGenLenti plasmid backbone (GenScript) and Invitrogen Virapower packaging plasmids in HEK293FT cells (Invitrogen R70007) and puromycin selection (2 μg/ml, Gibco). Tagged-factor integration was confirmed by Western blot with anti-FLAG antibody (#14793, D6W5B, Cell Signaling).

### Plasmid libraries

snapELISA screening plasmids used in this study were cloned into the pcDNA3.1 destination vector backbone using high-throughput Gateway recombination using pENTRY plasmids available within the human ORFeome7.1 and 8.1 libraries (38). In addition, a few pENTRY plasmids were generated by PCR and Gateway cloning using plasmids from the PlasmidID or synthesized products as PCR templates. pENTRY and final constructs were validated using restriction digestion (BsrGI, NEB) and Sanger sequencing.

Factors for TFO triplex assay were previously described in our prior study (18). The larger 520-factor library was generated for this study. A comprehensive description of factors and attributes can be found in Supplementary Table S2.

Plasmids for N-terminal-tagged validation experiments were custom synthesized by GenScript and cloned into a pCMV-3tag-1a backbone into the BamHI/XhoI restriction sites.

### Native-PAGE gel analyses

For native-PAGE analysis experiments, a photocleavable biotin conjugate (IDT, 5′ PC Biotin) was used to anchor and anneal structures on streptavidin-coated 96-well plates. At relevant time points, plates were washed, 20 μl of snapELISA buffer was added to each well, and plates were exposed to long-wave UV light (300–350 nm) using a handheld UV lamp for 10 min Five microliters of cleaved structures was loaded onto 5% TBE polyacrylamide gels (BIO-RAD) with BlueJuice loading buffer (Invitrogen) and run at 10 mA. Gels were stained with SYBR Gold (Invitrogen) and imaged using BIORAD GelDoc XRS+ by standard UV transillumination.

### PNA synthesis and delivery

γ-modified tail clamp PNA oligomers (IVS2 ^MP^γtcPNA, Supplementary Table S1) were synthesized manually on 10% lysine-loaded solid support (4-methylbenzhydrylamine resin, Peptides International, RMB-1045-PI) using standard Boc chemistry procedures. All Boc-aeg-PNA monomers were purchased from ASM Research Chemicals GmbH (Hannover). All ^MP^γPNA monomers were prepared from Boc-(2-(2-methoxyethoxy)ethyl)-L-serine as a starting material by a series of multistep synthetic procedures including reduction, mitsunobu reaction, nucleobase (A,C,G, and T) conjugation, and then ester cleavage. Kaiser tests were performed to ensure complete deprotection and coupling during each cycle. The oligomers were cleaved from the resin using a m-cresol:thioanisole:trifluoromethanesulfonic acid:trifluoroacetic acid (TFA) (1:1:2:6) cocktail solution (30 min x 2). The resulting mixtures were combined, and the crude PNAs were precipitated with cold ether, purified, and characterized by reverse-phase high-performance liquid chromatography (RP-HPLC) (5–95% ACN/water/0.1% TFA gradient) and MALDI-TOF spectroscopy (MALDI-TOF-MS Shimadzu AXIMA Confidence), respectively. See Supplementary Figure S2 for MALDI-TOF data from this study.

PNA stock solutions were prepared using Nanopure water, and the concentrations were determined using a Thermo Scientific NanoDrop OneC microvolume spectrophotometer. The following extinction coefficients were used: 13,700 M-1cm-1 (A), 6600 M-1cm-1 (C), 11,700 M-1cm-1 (G), and 8600 M-1cm-1 (T). PNAs were synthesized with 3 lysine (K) residues on N- and C-termini to facilitate solubility.

HPLC instrument set up consisted of the following: Waters 2998 Photodiode Array Detector, Waters 2545 Quaternary Gradient Module, Waters 2707 Autosampler. See Supplementary Figure S3 for HPLC tracing data from this study.

For PNA treatments in cells, 1 × 10^6^ K562 cells and 1 μl of PNA diluted in water to 200 μM (200 pmol total) were suspended in 100 μl of Lonza SF cell line solution (V4XC-2024, Lonza) and nucleofected using a Lonza 4D-Nucleofector X unit according to the manufacturer's instruction.

### snapELISA

snapELISA experiments were conducted on 96- and 384-well streptavidin precoated plates (Thermo Scientific REF#15502 & REF#15505). snapELISA buffer was composed of freshly prepared and filtered solutions of 50 mM Hepes-KOH (pH 7.9), 150 mM NaCl, 10 mM MgCl_2_, 0.7% Triton X-100, and 5% glycerol.

#### DNA triplex structure formation

DNA triplexes were formed by incubating 200 nM of biotin-conjugated duplex DNA and an equal volume of 400 nM AG30 TFO in snapELISA buffer for 30 min at 37°C. One hundred microliters (1 pmol) of the resulting structures was added to streptavidin-coated plates to incubate with rocking at room temperature for 30 min. Duplex DNA was formed from complementary 84mer ssDNA oligomers with 3 phosphorothioate linkages on either end and a 5′ biotin conjugate on one of the oligomers. AG30 TFO oligonucleotides featured C-6 amino (5′ end) and amino-c7 (3′ end) modifications to prevent nuclease degradation. Oligo sequences and modifications are summarized in Supplementary Table S1.

#### PNA heterotriplex structure formation

First, 100 μl of 100 nM target 5′ biotinylated ssDNA oligonucleotide (IDT) in snapELISA buffer was anchored to streptavidin-coated plates with rocking at room temperature for 30 min. Plates were washed 3 times with snapELISA buffer. One hundred microliters of 200 nM γMP-tcPNA was then added to wells and incubated overnight at 37°C. The following day, plates were washed 3 times with snapELISA buffer and 100 μl of 200 nM complementary ssDNA oligo (IDT) was added for another overnight incubation at 37°C. Oligo and PNA sequences and modifications are summarized in Supplementary Table S1.

#### Lysate preparation

For each corresponding structure-bound well, 10,000 HEK293T cells were separately transfected with 50 ng of 3× FLAG-tagged library plasmids using Lipofectamine 2000 (Invitrogen) in 96-well tissue culture plates in quadruplicated. Forty-eight hours later, wells containing cells were washed with PBS and 125 μl of snapELISA buffer (with supplemented RNAseA (Sigma), EDTA-free complete protease inhibitor, and 1 mM sodium vanadate, NEB) weree added and mixed for 5 min to induce cell lysis. Plates containing lysates were placed at 4°C for up to 30 min until needed to incubate with nucleic acid structures. Two of the replicate lysate plates were incubated with the triplex structure and the other two with duplex controls. Each plate included GFP (negative control), RPA2 (positive control), and XPA (positive control).

#### ELISA

Structure-bound streptavidin-coated 96-well plates were washed 3 times with snapELISA buffer and blocked using 100 μl of PBS with 1% BSA, 10 mM MgCl_2_ for 1 h rocking at room temperature. Wells were washed 3 more times, and 100 μl of the prepared HEK293T whole-cell lysate was removed from tissue culture plates and placed directly onto structure-bound wells. Lysate-containing wells were incubated at 4°C for 3 hours with rocking. Plates were then washed 5 times with snapELISA buffer and 100 μl of anti-FLAG horseradish peroxidase mouse Ab (A8592-.2MG, Sigma-Aldrich) diluted 1:20,000 in PBS with 1% BSA and 10 mM MgCl_2_ was added to incubate with rocking for 1.5 h at room temperature. Plates were finally washed 5 more times with PBST with 10 mM MgCl_2_ before adding SuperSignal West7014 Pico PLUS Chemiluminescent Substrate (Thermo Scientific, 34577) and reading plate luminescence using a Synergy H1 Multi-Mode Microplate Reader (Biotek).

#### High-throughput snapELISA (384-well)

For a high-throughput 384-well protocol, we used the Biotek EL406 automated plate washer and the Tecan EVO150 liquid hander robot, as described in our previous paper (39). This paper also describes the PEIMAX high-throughput transfection details for HEK293T cells.

### snapELISA data analysis and normalization

Raw luminescence data from each well were log2 transformed, and the derived values were used to calculate z-scores using mean background signal (empty wells) and the mean background standard deviation. This analysis was performed for both triplex and duplex wells. All PNA-triplex z-scores ≥ 5 were deemed significant. Next, preferential binding to PNA triplex structures was determined by calculating the difference between z-scores from corresponding triplex and duplex wells (Δz). To narrow down the list of most significant hits, z-score ≥ 5 and Δz ≥ 2.5 was set as the cutoff. Finally, data were sorted according to fold change of PNA-triplex binding over duplex binding. (See detailed analysis results in Supplementary Table S2).

### ChIP-qPCR

PNA-treated or PNA-untreated K562 cells (4 × 10^6^ cells) were diluted to 0.5 × 10^6^ cells/ml and cross-linked with a final concentration of 1% formaldehyde (Sigma-Aldrich, 252549) for each IP. Chromatin was prepared and sheared according to manufacturer protocol using SimpleChIP Enzymatic Chromatin IP Kit (Cell Signaling, #9003) and QSONICA Q800R3 sonicator for nuclear lysis. For each condition, 10 μg of chromatin was incubated with 10 μg of anti-FLAG antibody (#14793, D6W5B, Cell Signaling) or 1 μg of normal rabbit polyclonal IgG control antibody (Cell Signaling, #2729) rotating overnight at 4°C. Chromatin was incubated with 30 μl of protein G magnetic beads (Cell Signaling, #70024) and washed, eluted, reverse cross-linked, and purified according to the manufacturer's protocol.

qPCR reactions were conducted in technical triplicate for each biological replicate for 2% input and IP samples using a StepOnePLUS Real-Time PCR System (Applied Biosystems), SimpleChIP Universal qPCR Mastermix (Cell Signaling, #88989), and HBB-IVS2 PNA target specific primers (Supplementary Table S1). Nontarget primer qPCR controls were also conducted for each replicate (Supplemental Fig. S7). Percent occupancy and fold enrichment values were calculated by percent input method from 2% input samples for each replicate.

### Statistics

Graphing and statistical analysis were performed for each dataset using GraphPad Prism 9 (v9.3.0) software unless otherwise mentioned. All relevant equations, differences, and *p*-values from this study are summarized in Supplementary Table S3. For Fig. 2, *B* and *C* and Fig. S6, comparisons were calculated using Dunnett’s multiple comparisons test after confirming significant two-way ANOVA interactions. For Fig. 3, *C* and *F*, Fig. 4*B*, and Fig. S9, an unpaired student’s *t* test was used to compare conditions for each group. For Fig. 2*D* and Fig. 3*D*, Fig. S1, and S5, a simple linear regression analysis was performed and used to determine best-fit line equation and R^2^ values. Illustrations were generated using BioRender.com software.

## Data availability

Detailed data and library information from snapELISA screen are available in Supplementary Table S2. Custom plasmid library used in this study is available via DNASU Plasmid Repository, submitted as “Genome Maintenance, 10 x 96-well plates” by the Karras Lab. All data are available from the corresponding authors upon reasonable request.

## Supporting information

This article contains supporting information.

## Conflict of interest

P. M. G. and N. G. E. are inventors on patents related to PNAs and gene editing that are assigned to Yale University. P. M. G. is a consultant to and has equity in Cybrexa Therapeutics, pHLIP Inc, and Gennao Bio and has an equity interest in Patrys Limited. None of these entities were involved in or are related to the work reported here.
