# Supplementary material for: An ELISA-based platform for rapid identification of structure-dependent nucleic acid–protein interactions detects novel DNA triplex interactors
Source: J Biol Chem. 2022 Aug 18;298(10):102398. doi: 10.1016/j.jbc.2022.102398 (PMC9493393; doi:10.1016/j.jbc.2022.102398)
Supplement: Supporting Information [file mmc1.pdf]

Supporting Information for

An ELISA-based platform for rapid identification of structure-dependent nucleic acid-protein interactions detects novel DNA triplex interactors

Nicholas G. Economos, Upasna Thapar, Nanda Balasubramanian, Georgios I. Karras*, and Peter M. Glazer*

*Correspondence to: GKarras@mdanderson.edu, peter.glazer@yale.edu

This PDF file includes:

Figures S1 to S9
Table S1

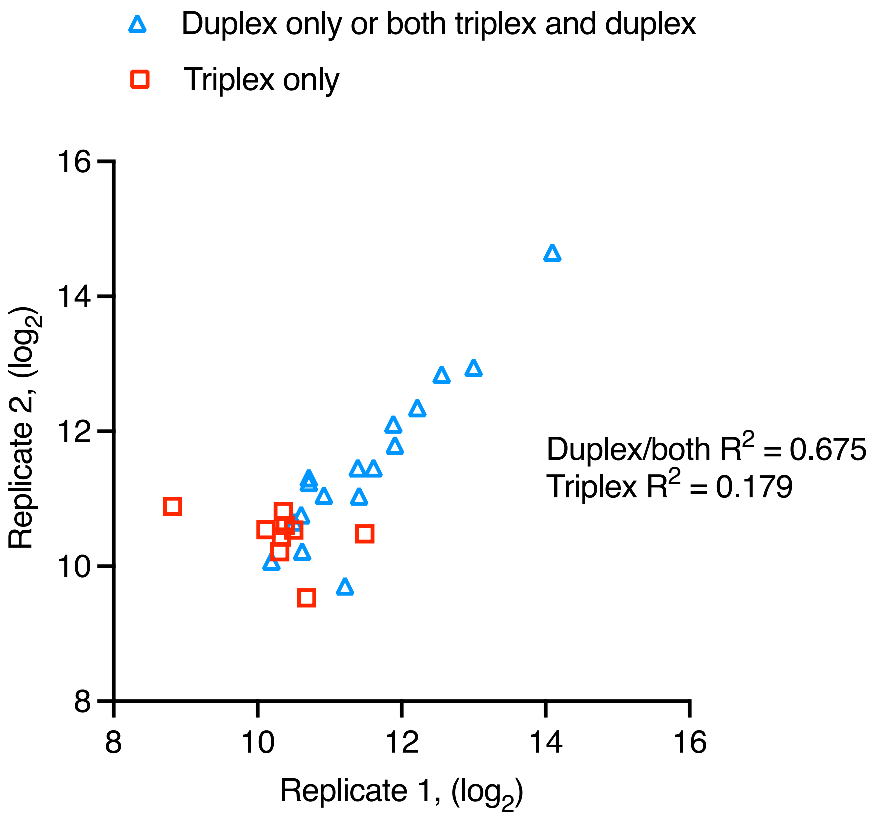

Figure S1. Fold-change (log2) values for significant replicates in TFO triplex screen. Values for significant duplex or duplex and triplex binders (blue) and TFO triplex binding (red) replicates are plotted. Binding significance was determined by z-score > 5. Fold-change (log2) is calculated relative to non-binding control on each plate. R^2 for both groups is labeled on plot.

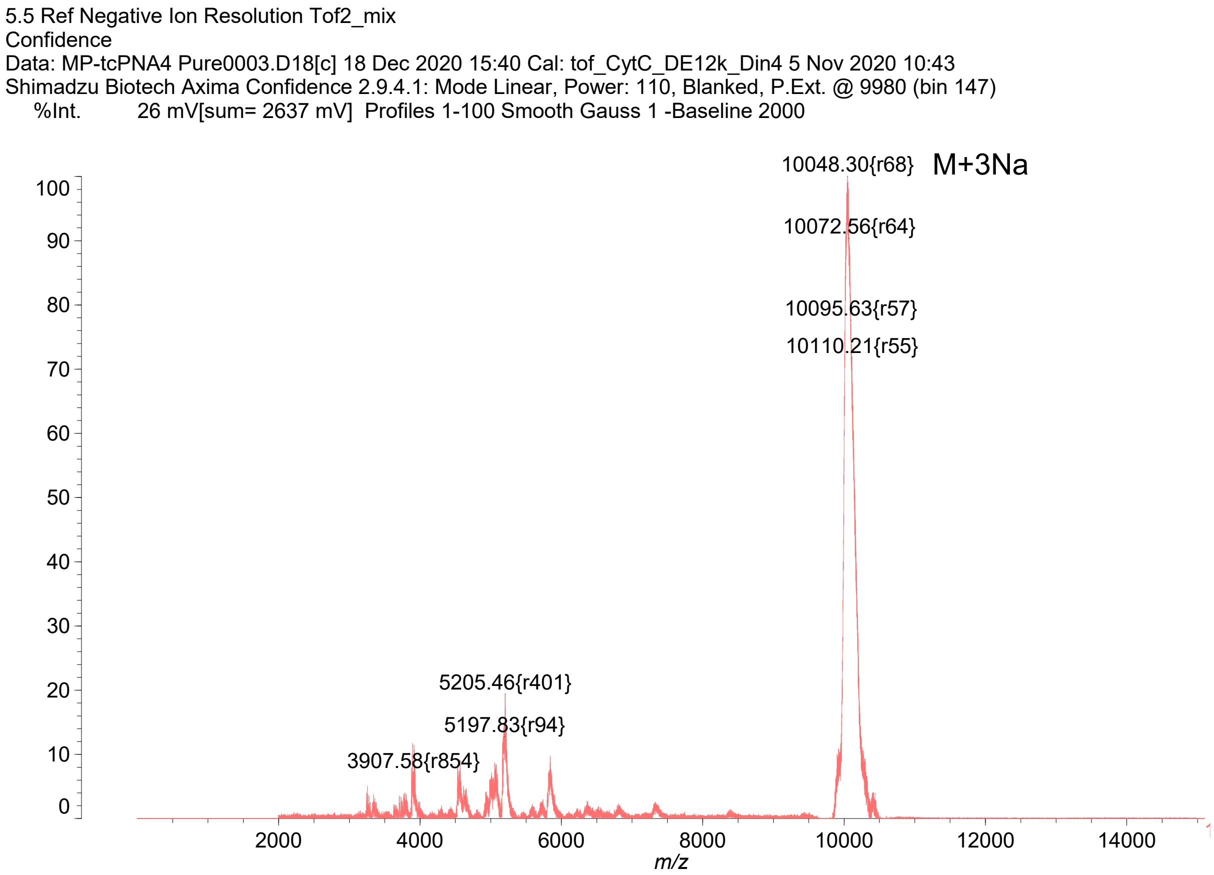

Figure S2. Mass spectroscopy analysis of purified γMP- tcPNA product used to generate PNA heterotriplex structures used in snapELISA and for ChIP-qPCR experiments. Expected mass was 9982 g/mol. Observed mass peak plus additional ions (M+3Na) is labeled.

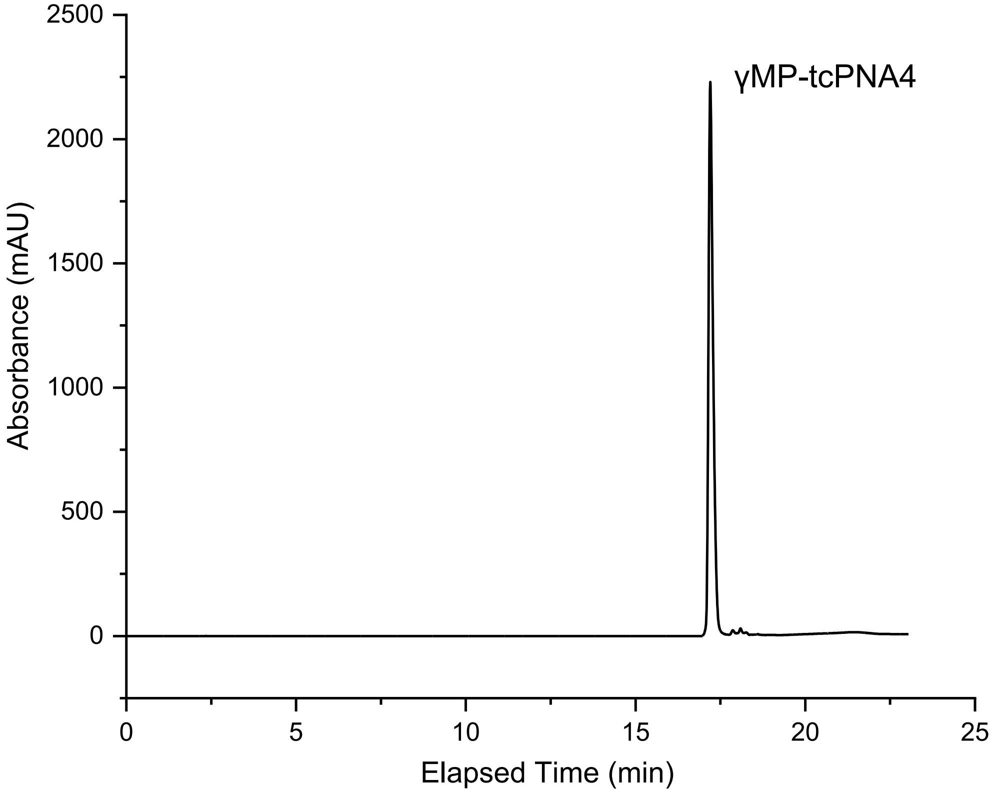

Figure S3. Purified γMP-tcPNA4 HPLC trace in 5-95% CAN/water, 0.1% TFA. Absorbance (260nm) was measured throughout experiment to detect the presence of nucleic acid products. A pure single peak indicating the product of interest is labeled on the plot.

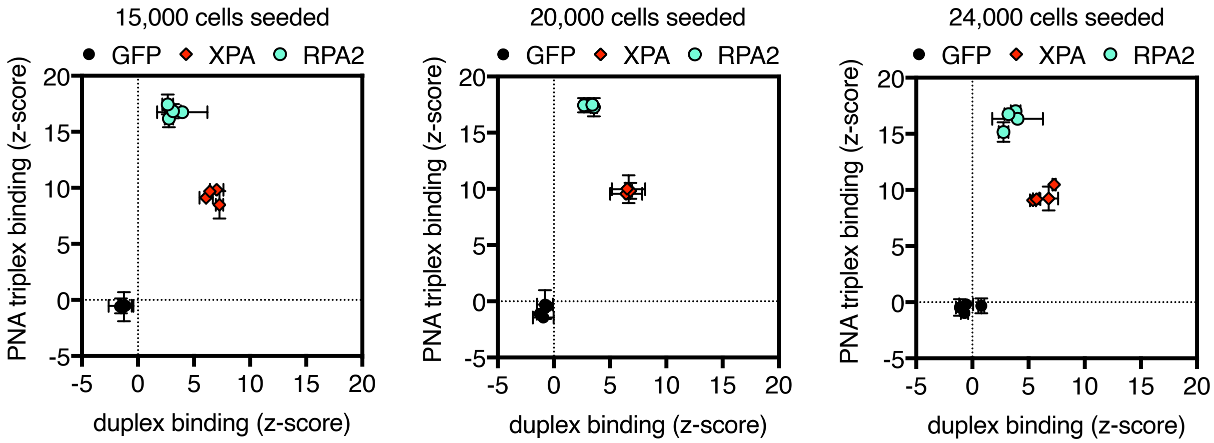

Figure S4. snapELISA validation experiments at additional transfection cell densities. The number of HEK293T cells seeded for transfection is 15,000, 20,000 or 24,000. Indicated expression constructs were transfected in quadruplicates and snapELISA binding assays were performed in technical duplicates against DNA duplex and PNA heterotriplex. Z-score averages and standard deviations from technical duplicates from *n*=4 independent experiments are shown for each condition.

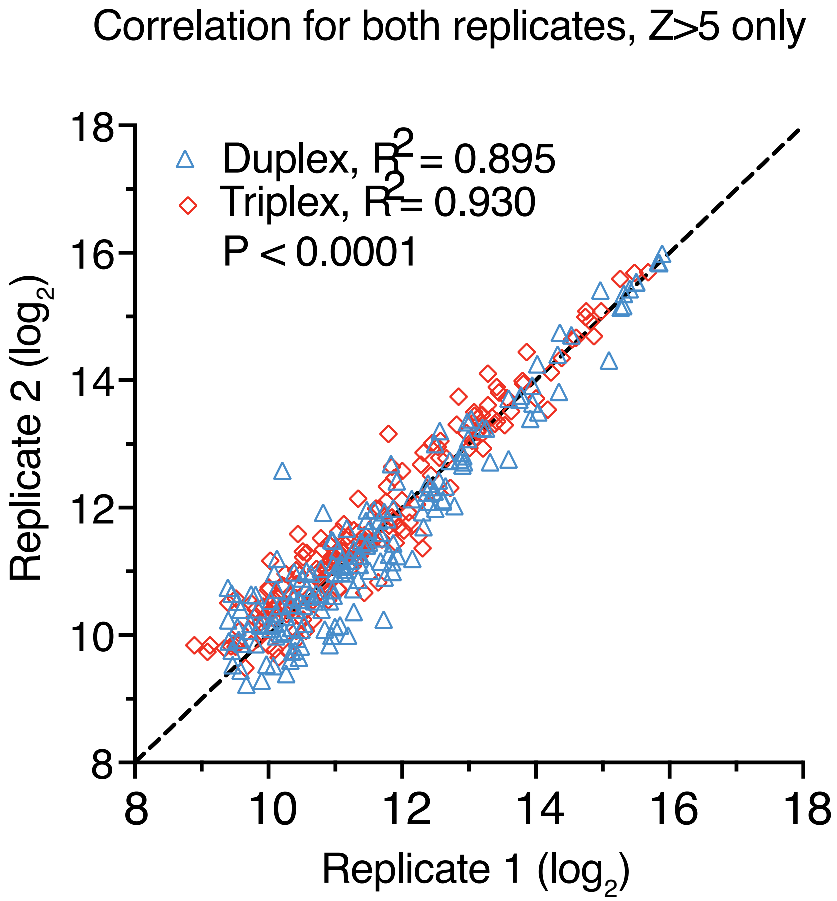

Figure S5. Fold-change (log2) values for significant replicates in PNA heterotriplex screen. Values for significant duplex (blue) and PNA heterotriplex binding (red) replicates are plotted. Binding significance was determined by z-score > 5. Fold-change (log2) is calculated relative to non-binding control on each plate. R^2 and p-value for both groups is labeled on plot.

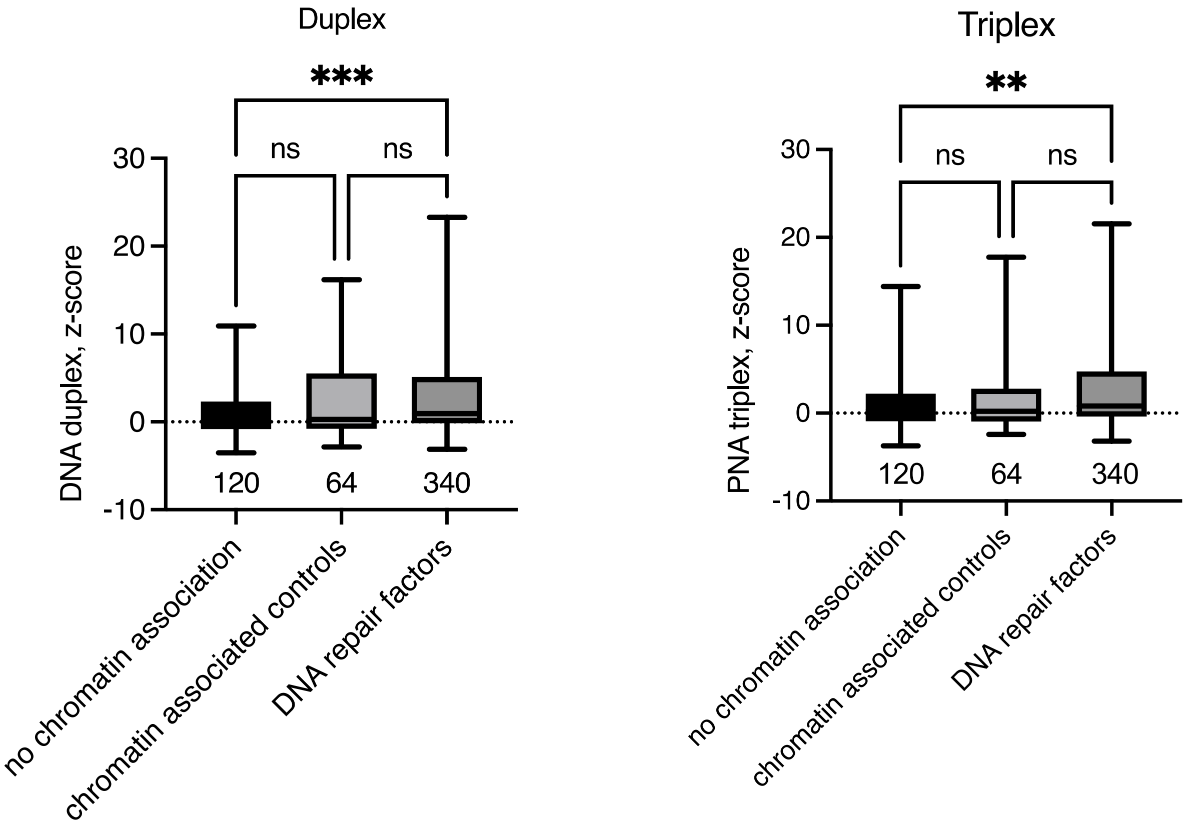

Figure S6. Z-scores for DNA duplex and PNA heterotriplex snapELISA screens organized by factor classification. Box-and-whisker plots represent mean with 25th and 75th percentiles and maximum and minimum values, p-values listed in Supplementary Table S3.

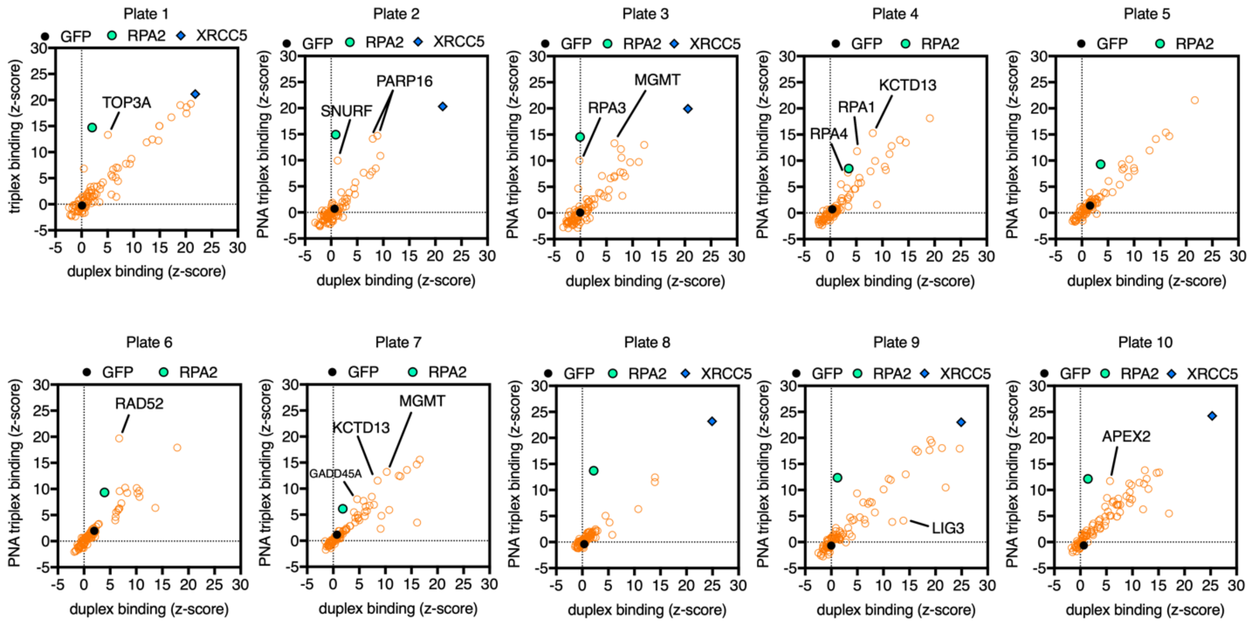

Figure S7. SnapELISA results by individual plate from heterotriplex PNA screen. Duplex and PNA heterotriplex binding z-scores from each experiment are plotted. Each plot represents an experiment on one plate, with GFP negative control (black) and RPA2 positive control (cyan) labeled. Select factors of interest are labeled on plots.

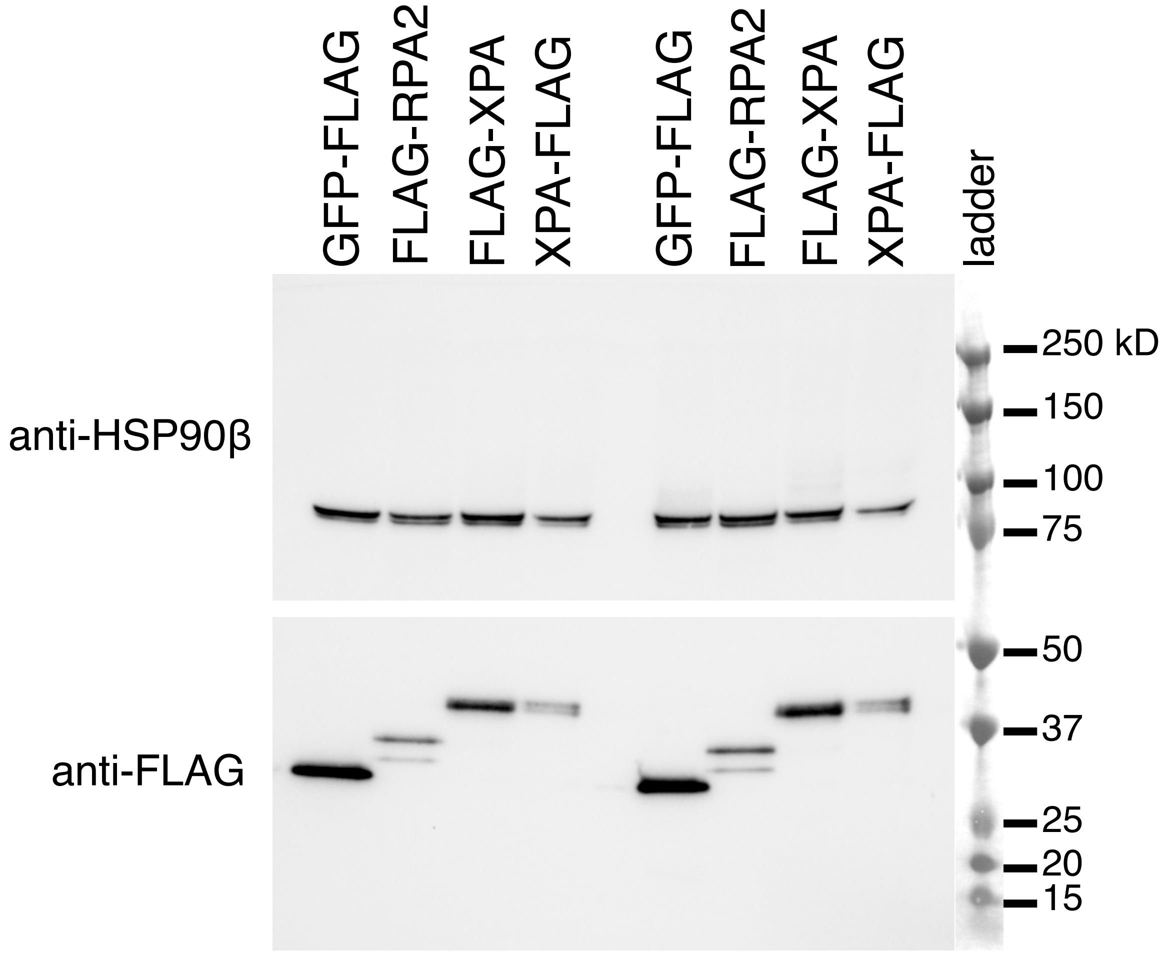

Figure S8. Western blot for select FLAG-tagged factors expressed in HEK293T cells. All factors are 3X FLAG-tagged. “FLAG-XPA” describes N-terminal 3XFLAG-tagged construct used in initial validation experiments (Fig. 3C), while “XPA-FLAG” describes C-terminal 3X-FLAG-tagged constructs used in larger-scale screen (Fig. 3E). Two replicates for each sample, HSP90beta loading controls, and reference molecular weight markers are shown.

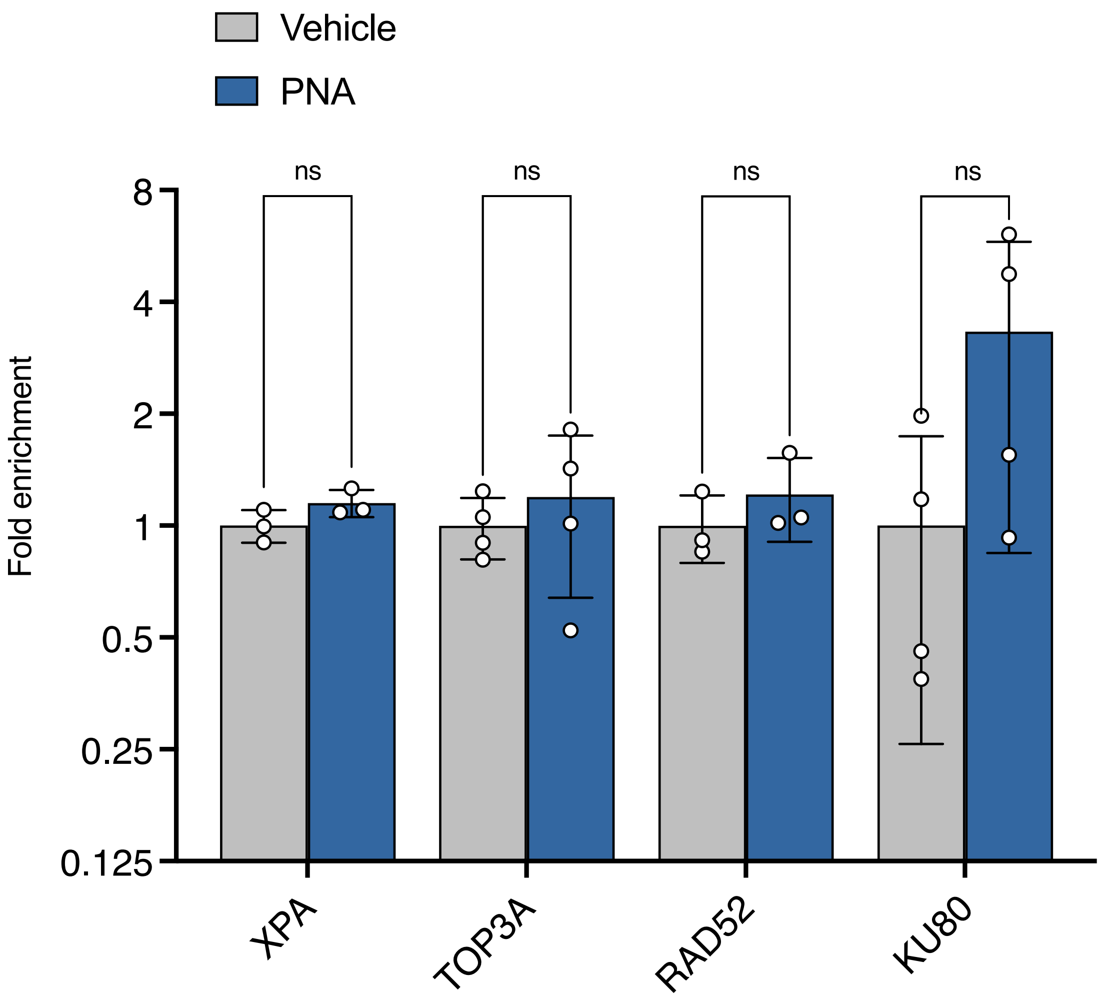

Figure S9. Fold-enrichment ChIP signal for 3X-FLAG-tagged factors in K562 lines. Experiments used α-FLAG antibody and non-targeting control qPCR target (Human RPL30 exon 3). Bars represent mean ± s.d. from at least *n*=3 independent experiments, p-values listed in Supplementary Table S3.

Oligo	Sequence	Notes	Source
IVS2 PNA target oligo	CAAAGAGGCATGATACATTGT ATCATTATTGCCCTGAAAGAAA GAGATTAGGGAAAGTATTAGA AATAAGATAAACAAAAAA	5' biotin (or pcBiotin) conjugate, 82mer, three phosphorothioate linkages on either end, PAGE purified	IDT
IVS2 complem ent oligo	TTTTTTGTTTATCTTATTTCTAA TACTTTCCCTAATCTCTTTCTT TCAGGGCAATAATGATACAAT GTATCATGCCTCTTTG	82mer, three phosphorothioate linkages on either end, PAGE purified	IDT
DNA triplex sense oligo	CTGAATTCCCGTCATCGACTT CGAAGGTTCGAATCCTTCCCC CCCCACCACCCCCTCCCCCTC GGCCGAAATTCGGTACCCGG AT	5' biotin conjugate, 84mer, three phosphorothioate linkages on either end, PAGE purified	IDT
DNA triplex antisense oligo	ATCCGGGTACCGAATTTCGGC CGAGGGGGAGGGGGTGGTG GGGGGGGAAGGATTCGAACC TTCGAAGTCGATGACGGGAAT TCAG	84mer, three phosphorothioate linkages on either end, PAGE purified	IDT
AG30 TFO	AGGAAGGGGGGGGTGGTGGG GGAGGGGGAG	30mer, C-6 amino (5’ end) and amino-c7 (3’ end) modifications, PAGE purified	IDT
**PNA**			
IVS2 ^{MP}γtcPNA	KKK-JTTTJTTTJTJT-000- T**C**T**C**T**T**T**C**T**T**T**C**A**G**G**G**C**A**-KKK	Bold/underline indicates γPNA residues; K indicates lysine, J, pseudoisocytosine, 0, 8-amino-2,6,10-trioxaoctanoic acid linkers connecting the Hoogsteen and Watson–Crick domains	Manually synthesized in-house
**Primers**			
IVS2.IP.F	ACAATCCAGCTACCATTCTGC T		In-house (Keck)
IVS2.IP. R	GCAAAAGGGCCTAGCTTGGA		In-house (Keck)
Non-targeting qPCR primers	SimpleChIP® Human RPL30 Exon 3 Primer set #7014 (161bp product)	Control primer set from SimpleChIP Enzymatic Chromatin IP Kit (Cell Signalling #9003)	Cell Signalling

Table S1. Oilgos, PNAs, and primers used in this study.
